# Understanding the challenges and successes of implementing ‘hybrid’ interventions in healthcare settings: findings from a process evaluation of a patient involvement trial

**DOI:** 10.1136/bmjqs-2024-017268

**Published:** 2024-08-06

**Authors:** Sarah Hampton, Jenni Murray, Rebecca Lawton, Laura Sheard

**Affiliations:** 1 Health Sciences, University of York, York, UK; 2 Yorkshire Quality and Safety Research Group, Bradford Institute for Health Research, Bradford, UK; 3 Institute of Psychological Sciences, University of Leeds, Leeds, UK

**Keywords:** Implementation science, Healthcare quality improvement, Health services research, Patient safety, Qualitative research

## Abstract

**Introduction:**

‘Hybrid’ interventions in which some intervention components are fixed across sites and others are flexible (locally created) are thought to allow for adaptation to the local context while maintaining fidelity. However, there is little evidence regarding the challenges and facilitators of implementing hybrid interventions. This paper reports on a process evaluation of a patient safety hybrid intervention called Your Care Needs You (YCNY). YCNY was tested in the Partners at Care Transitions (PACT) randomised controlled trial and aimed to enhance older patients and their families’ involvement in their care in order to achieve safer transitions from hospital to home.

**Methods:**

The process evaluation took place across eight intervention wards taking part in the PACT trial. 23 interviews and 37 informal conversations were conducted with National Health Service (NHS) staff. Patients (n=19) were interviewed twice, once in hospital and once after discharge. Interviews with staff and patients concerned the delivery and experiences of YCNY. Ethnographic observations (n=81 hours) of relevant activities (eg, multidisciplinary team meetings, handovers, etc) were undertaken.

**Results:**

The main finding relates to how staff understood and engaged with YCNY, which then had a major influence on its implementation. While staff broadly valued the aims of YCNY, staff from seven out of the eight wards taking part in the process evaluation enacted YCNY in a mostly task-based manner. YCNY implementation often became a hurried activity which concentrated on delivering fixed intervention components rather than a catalyst for culture change around patient involvement. Factors such as understaffing, constraints on staff time and the COVID-19 pandemic contributed towards a ‘taskification’ of intervention delivery, which meant staff often did not have capacity to creatively devise flexible intervention components. However, one ward with a sense of distributed ownership of YCNY had considerable success implementing flexible components.

**Discussion:**

Hybrid interventions may allow aspects of an intervention to be adapted to the local context. However, the current constrained and pressured environment of the NHS left staff with little ability to creatively engage with devising flexible intervention components, despite recognising the need for and being motivated to deliver the intervention.

**WHAT IS ALREADY KNOWN ON THIS TOPIC**

- Healthcare interventions sometimes comprise both fixed components that are the same across sites and flexible components that are specific to the local context. However, there is little evidence exploring how these interventions are implemented in practice in the field of patient safety.

**WHAT THIS STUDY ADDS**

- This process evaluation of a patient safety intervention suggests that flexible intervention components may bring benefits though are difficult to implement within pressured healthcare settings.

**HOW THIS STUDY MIGHT AFFECT RESEARCH, PRACTICE OR POLICY**

- Those developing healthcare interventions may wish to consider that flexible components are challenging to implement in a pressured healthcare context without strong leadership, a team-wide approach and additional support to bolster the role of healthcare staff.

## Introduction

 Healthcare interventions have historically been standardised according to form across sites, such that each site delivers the same intervention components in the same manner.^1^ This approach allows for fidelity (the extent to which the intervention is delivered as intended) to be maintained across sites. However, there is increasing acknowledgement of the role of local context in determining how an intervention is implemented and its effects.^2^ To allow for adaptation to local context while retaining fidelity, it has been suggested that interventions should be standardised across sites according to the function (aim) of the components rather than their form.^3 4^

A further interpretation of this approach is the current trend towards ‘hybrid’ interventions in which some intervention components are fixed across sites and others are flexible.^1^ Flexible components are those that are defined by designers who prescribe the function but whose form is devised by staff at each site according to what works for their context. Flexible components allow the intervention to be tailored to the setting in which it is implemented and can offer greater autonomy to those implementing the intervention. There is some evidence that frontline staff can be resistant to change when it is perceived as externally led.^5^ As such, cultivating a sense of ownership over the design of an intervention among frontline staff via flexible components may facilitate intervention implementation. However, frontline staff’s engagement in an intervention is highly variable and influenced by competing clinical demands, understaffing and inadequate resources.^5 6^ Furthermore, nursing and other frontline healthcare work is often broken down into routine, repetitive tasks with little space for creative thinking and flexibility (known as ‘taskification’).^7^ Frontline staff may lack the time, resources and engagement to contribute creatively to producing the flexible components of an intervention. As such, while hybrid interventions may ideologically bring benefits, including context sensitivity and local ownership, the extent to which hybrid interventions achieve these functions in practice requires further exploration.

The Your Care Needs You (YCNY) intervention is a hybrid patient safety intervention supporting older patients and their families to be more involved in their care in hospital to prepare them for safely reassuming aspects of their care after discharge.^8 9^ This hybrid approach was considered appropriate as YCNY is designed to be delivered across any context (ie, all ward types) where care for older people is provided. Patients and families are often a source of variability within healthcare systems. While this variability has been thought as unwanted variation, patients and families may be able to ‘reach in’ (proactively take on responsibility for managing aspects of their care) and contribute to system resilience (such as by chasing up providers in response to delays in scheduling appointments and proactively contacting their general practitioner following hospital discharge when medications have been changed).^10^ This is in line with conceptualisations of healthcare as a complex system that is constantly adapting to changes in unpredictable circumstances (resilient healthcare theory^11^). Patient/family involvement is especially important during transitions from hospital to home. Transitions can pose risks, with approximately one in five patients experiencing an adverse event, such as a medication error, during this time.^12^ Transitions can be especially risky for older patients, with 30-day hospital readmission rates for patients over 75 years old estimated at around 16%.^13^ Transitions represent a handover of care from the hospital to patients/families, with patients/families taking on responsibilities at home that were performed by staff in hospital.^14^ This handover of care is often not formally acknowledged, and staff consequently may not prepare patients adequately for this handover. As such, involving patients and families in their care in hospital may help to prepare them to take over aspects of their care after discharge and ultimately reduce avoidable hospital readmissions and enhance the quality and safety of their care.^15^

This paper reports the findings of the qualitative process evaluation of YCNY being tested in a randomised controlled trial (the Partners at Care Transitions (PACT) trial). The paper focuses on how staff and patients understood the intervention and how this influences staff implementation of it, in addition to the contextual factors that enable and impede implementation. Central to this is an examination of fixed and flexible components in hybrid interventions and their role within capacity pressured healthcare systems.

## Methods

### The PACT trial and YCNY intervention

The process evaluation ran alongside the PACT cluster randomised controlled trial,^16^ which assessed the effectiveness of YCNY for patients aged 75 years and over. The trial ran across 39 hospital wards in 11 trusts (organisational units within the National Health Service (NHS) providing secondary care within a geographical area) and randomisation occurred at ward level.

YCNY aims to support older patients to know more and do more in four key areas (functions): (1) managing health and well-being, (2) managing medications, (3) completing daily activities (eg, mobilising) and (4) anticipating needs and escalating care. These four functions were determined by applying the functional resonance analysis method to qualitative data regarding the pathways involved in care transitions.^10^ The programme theory of change suggested that patients’ ability to carry out these functions at home would be influenced by their involvement in these functions in hospital. As such, the intervention aims to support patient involvement in these key areas. YCNY aims to empower patients and families to ‘reach in’ where there are gaps in the healthcare system, aiding system resilience.^13^ YCNY therefore aims to promote a culture of patient involvement, moving away from a culture in which the role of staff is to undertake activities on behalf of the patient.

YCNY involves fixed and flexible components. The fixed components comprise patient-facing materials, including a booklet, advice sheet and video which patients receive in hospital. These components were aimed at patients and families and were codesigned with patients and staff. These components were chosen to be tangible, easy-to-use resources that could help patients ‘reach in’. When choosing the format of the components, several factors were considered, including: the extent to which it supported patient involvement, whether it could meet all of the intended functional aims, whether it could facilitate communication between staff and patients and its practical implementation.

The booklet explains how patients can be more involved in the four key functions and represents a tool to enhance communication with staff by suggesting questions patients could ask staff and providing space to write questions. The booklet is given to patients alongside a brief explanation. The advice sheet includes information on how to escalate care when home. The video depicts older people talking about their experience in hospital and home, focusing on the four functions. YCNY materials also include patient-facing posters giving advice on the four functions that staff are encouraged to adapt to their ward. Staff are also encouraged to devise flexible components to enhance patient involvement in the four functions. YCNY was aimed at all staff types, and the roles of different staff types were not prescribed by the research team but decided by staff on each individual ward. Senior staff members (eg, managers, clinical leads) were engaged in the trial set-up and encouraged to join a YCNY training session (see below) and to play a supporting role throughout intervention delivery.

Staff acting as facilitators on each ward attended a YCNY training session run by the PACT team to introduce the theory and practicalities of YCNY. Staff were encouraged to tailor YCNY to the context of their ward and were provided with examples of flexible components, such as using ‘teachback’ methods to promote patients’ understanding of their medications, and encouraging patients to get dressed. Trained staff members were then responsible for cascade training of other staff members on the ward. Staff were also provided with access to a website with resources to aid YCNY implementation.

### Process evaluation design, sample and data collection

Data were collected at eight intervention wards across four trusts taking part in the PACT trial (details in table 1). Trusts were chosen to achieve variation in geography, trust size, ward specialty and affluence/deprivation of the geographical area. Data consisted of staff interviews (n=23), patient interviews (n=19) and ethnographic observations. Interviews with families/carers were also intended to take place, though recruitment issues (including reduced visiting hours during the COVID-19 pandemic) meant that this was not possible. Interviews were audio recorded and transcribed, otherwise detailed notes were taken. All interview participants gave written or witnessed informed consent.

**Table 1:** Data collected for each trust

	Trust pseudonym				
	Cedar	Oak	Birch	Elm	Total
Wards, n	3	2	2	1	8
Specialty	2 intermediate care;1 vascular	2 elderly care	1 elderly care;1 acute abdominal medicine and surgery	Haematology, neurology, oncology and stroke	–
Staff members who attended YCNY training	Cedar 1: ward manager, healthcare assistant, rehabilitation assistantCedar 2: two nurses, two rehabilitation support workersCedar 3: three nurses, nurse associate, deputy matron	Oak 1: two nurses, healthcare assistant, three occupational therapistsOak 2: Principal Investigator, matron, two nurses, pharmacist, physiotherapist	Birch 1: three clinical support workersBirch 2: eight nurses, discharge coordinator, care support worker, ward manager	Principal Investigator, ward manager, stroke practitioner, advanced care practitioner, occupational therapist, physiotherapist, care support worker, nurse	
Total hours of ethnographic fieldwork	32	16	20	13	81
Patient interviews	9	6	4	0	19
Staff interviews	7	5	7	4	23
Informal conversations with staff	19	8	7	3	37

Note. Names of trusts are pseudonyms. Each trust is denoted by the name of a tree (chosen at random).

YCNYYour Care Needs You

Patients were recruited while resident in hospital wards where the trial was taking place. Participants were eligible if they were aged at least 75 years and due to be discharged back to their own/a relative’s home. Purposive sampling was conducted in order to achieve variety in gender and ages over 75 years. Patient age and gender can be found in table 2. All participants were of white ethnicity. Two semistructured interviews were conducted with each participant. The first took place while the patient was in hospital and the second via telephone between 6 and 11 days after discharge. Interviews concerned patients’ experiences of the four YCNY areas and their perceptions of YCNY (see online supplemental material). Participants were given a £20 gift voucher. Patient data were not collected on one ward (Elm). Recruitment was challenging on Elm as the ward changed specialty and most staff involved in YCNY left the ward. The findings reported from this ward refer only to prior to the ward changing specialty.

**Table 2:** Patient demographic data for each ward

	Cedar	Oak	Birch	Elm	All
Mean age in years (range)	83.10 (78–89)	88.80 (83–95)	82.54 (77–84)	n/a	84.78 (77–95)
Gender (female:male)	5:4	2:4	2:2	n/a	9:10

Semistructured interviews were conducted with 23 staff involved in the YCNY intervention. Interviews concerned how YCNY was implemented and the challenges and facilitators of implementation. Staff roles included ward managers, nurses, discharge coordinators, clinical support workers, physiotherapists and occupational therapists. Staff varied in their professional experience (mean years since qualifying was 13.31, range 1–28 years) and time on the ward (mean 5.52 years, range 1–15 years). The interviews predominantly took place on the ward with a minority via telephone.

81 hours of ethnographic fieldwork (excluding formal interviews but including observations and informal conversations) were conducted by the first author. Four to six visits were conducted per ward over a period of 2–4 months in order to conduct interviews with staff and patients, to have informal conversations with staff and to carry out general ward observations and observations of activities relevant to YCNY (eg, multidisciplinary team meetings, handovers, medication rounds and patient exercise classes). Visits were intended to take place over the whole 4-month intervention period; however, due to recruitment issues for the main trial wards sometimes implemented YCNY for more than 4 months and as such the process evaluation did not always capture the entire intervention period. Written informed consent for the observations was given by a senior member of staff on each ward. Verbal consent was given by staff and patients involved in any activity being observed. Detailed fieldnotes were written for each visit. Data collection took place between February 2022 and March 2023.

Additional contextual data were collected including researchers’ notes from the YCNY staff training sessions, notes taken by research nurses during recruitment and interviews/focus groups (n=6) with the PACT team concerning their tacit knowledge of the trusts during study set-up.

### Data analysis

Data were analysed by the first and last authors using a constant comparison approach.^17^ After data collection was complete for the first trust, both authors read through the data (fieldnotes, transcripts and notes by the PACT team and research nurses) and independently devised themes. Through discussion, the two authors reached consensus on and refined the themes for that trust. For each trust after the first, the themes and subthemes for that trust were compared with those of the trusts previously analysed. Once data collection for all trusts was complete, the two authors met to reach consensus on the overall themes across trusts. At each stage, the themes and subthemes independently devised by the two authors were almost identical. The first author then returned to the dataset to undertake further interpretation and sense checked with the last author iteratively as themes were written up. All authors approached the data from the perspective of applied qualitative healthcare researchers.

## Results

Three themes were devised: (1) suitability of the intervention for the patients and ward, (2) wider macro context (ie, trust-wide context) and (3) understanding the ethos of the intervention. Theme 1 refers to the challenges of finding patients who are able to engage with the intervention but not so independent as to derive little use from it. Theme 2 describes the impact of wider organisational factors such as staffing and trust-wide policies. As the focus of this paper is on the implementation of hybrid interventions across different contexts, we choose to focus on the final theme, understanding the ethos of the intervention, in this paper. Themes arising from PACT but not detailed in this paper have been reported elsewhere.^18^

### Understanding the ethos of the intervention

This theme, comprising three subthemes, explores how staff and patients understood the intervention and how this shaped its implementation.

#### Staff’s understanding of the intervention and their implementation of it

Most staff recognised and valued the core aims of YCNY, namely to achieve safe transitions and avoid readmissions. Staff generally recognised that hospital readmissions are sometimes avoidable and that preparing patients for going home safely may help avoid unnecessary readmissions. Some staff (particularly at Elm and Birch) additionally understood and valued the role of patient involvement in achieving safe transitions.

For me it’s kind of trying to get patients to take back control a bit of everything that we take away from them which is their independence isn’t it, and allowing them to have a voice to speak up and ask questions. (Ward manager, Elm)

Further, some wards felt the focus on patient involvement fitted well with the ethos of their ward. Wards focusing on rehabilitation (Cedar, Elm, Birch 2) were particularly able to connect with the focus on patient mobility, recognising that encouraging patients to independently complete physical tasks is important for rehabilitation. These wards were more likely to engage therapy staff in YCNY delivery and to direct the intervention towards their pre-existing goals around encouraging patient mobility.

On the admission, we implicate to [the patient’s] mind that okay, you are here for rehabilitation, you have to do something for yourself, you have to help yourself. (Senior nurse, Cedar 1)

However, the relevance of patient involvement was not recognised by all staff. One site (Oak) perceived YCNY to target staff errors around discharge rather than patient involvement. Understanding, and therefore implementation, of YCNY at Oak was further complicated by confusion between what was part of the intervention and what was a trial process (despite the YCNY training instructing staff to disregard research processes). The roles of the research nurses and ward staff had become muddled on Oak wards, meaning that ward staff were not able to fully take responsibility for delivery of YCNY nor appropriately tailor YCNY to their particular ward’s needs.

I wasn’t expecting any sort of like nurse involvement in it whatsoever. Which in hindsight was a bit naïve of me but, but then obviously research [nurses] took it on anyway didn’t they? (Ward manager, Oak 2)

#### Delivering fixed components versus developing and implementing ward-specific flexible components

Staff on all wards reported giving out the booklets and advice sheets to patients (this was sometimes done via an admission pack), and all wards displayed YCNY posters. For most wards, delivering the fixed components became their main focus for YCNY. Together with constraints on staff time due to understaffing and COVID-19-related pressures, this meant that YCNY could become a task-based activity of giving out the materials rather than creatively tailoring components to local issues.

Despite this focus on fixed components, most patients interviewed did not recall receiving a booklet or advice sheet, perhaps indicating relatively low delivery or low recall of having received the materials. Booklets were rarely visible in patients’ rooms and patients/families were not seen interacting with the booklet during observations. Patients who received a booklet sometimes reported storing it away with other paperwork, perhaps indicating that patients did not view the booklet as a tool to prompt care interactions. Some patients tended to view YCNY as about receiving information rather than a tool to support involvement, ‘*So yeah, really a lot of it was fairly basic information*’ (Patient, 86-year-old woman, Cedar 2). Patients often considered themselves to be active and independent, with a reasonable understanding of their health. Perhaps due to perceiving the YCNY materials as information rather than tools to assist involvement, these patients tended to find YCNY materials too basic to be personally relevant. It is unclear to what extent patients’ self-perception as active and knowledgeable was accurate, though patients were generally able to describe their medications and daily activities in some amount of detail.

I’m pretty, well genned up about the doctors and everything […] if they’d have gone into more detail I think it would have been more helpful for me. (Patient, 78-year-old woman, Cedar 1)

Staff often did not have the time or capacity to devise flexible intervention components. Some wards were able to make small changes (eg, explaining medicines to patients in more detail) but these were often limited to the enthusiasm of one or two staff members rather than a ward-wide approach (Cedar, Birch 1). However, one ward, Birch 2, had considerable success in implementing flexible components. They introduced an exercise class led by a physiotherapy assistant, ‘*We’re doing a weekly exercise class which we’ve had a really good response from and all the patients have really enjoyed*’ (Allied health professional, Birch 2). The ward also introduced an exercise sheet to give to patients to enable them to exercise independently, *‘The physio gave me a series of exercises and I’ve been doing those. And I’ve been walking round, I’ve just started independently with the frame walking round the, walking round the ward*’ (Patient, 77-year-old man, Birch 2). This focus on flexible components on Birch 2 seemed to be associated with a mindset of broader culture change around patient involvement for some staff, ‘*[The ward manager] talked about culture change being important, and embedding YCNY into usual practice. She seemed more focused on the flexible components rather than the fixed components*’ (Fieldnotes, Birch 2).

#### Ownership of the intervention

Ward-wide, distributed understanding of YCNY was often not present, with responsibility for implementing YCNY sometimes limited to one or two staff members. Some wards had an incredibly dedicated ward facilitator, often a senior nurse, who took the lead with other staff playing a supporting role. While this approach could be advantageous, for some wards it could mean a lack of knowledge of YCNY filtering through the ward, *‘I did have 2 weeks off in December as well and I think if we aren’t in then it kind of gets forgotten about a little bit’* (Ward manager, Cedar 3). Shift work and high staff turnover, particularly among non-nursing staff, could mean that few staff knowledgeable about YCNY were present on the ward at any one time, impeding a ward-wide approach, *‘It’s just quite a lot on the nurses to do I felt’* (Nurse, Oak 2).

On Elm, ownership of YCNY seemed to fall between the nursing and therapy teams, with each team expecting the other to take the lead, resulting in neither team truly taking ownership.

Thinking about the therapy team, because we haven’t felt like we’ve had a lot of ownership over it its perhaps not something that we actively seek to question about. (Occupational therapist, Elm)

For Birch 2, however, knowledge of YCNY was distributed throughout the ward. This was perhaps due to a high number of staff having attended the YCNY training, under the request of the ward manager. This multidisciplinary team (MDT) approach seemed to facilitate deeper understanding of YCNY throughout the ward.

I think if it’s going to work you need all the MDT involved and not just nurses on the ward really, I think it needs to involve like your pharmacists, it needs to involve your physios, you know discharge coordinators. (Nurse, Birch 2)

## Discussion

The process evaluation found that staff valued the aims of YCNY. However, pressures on staff’s time meant that they could struggle to develop and implement the more creative, flexible components and as such YCNY could become a task-based endeavour of delivering the fixed materials. Delivery of these materials could further become a hurried exercise with little time to explain the materials to patients. Patients tended to engage with the materials as ‘information’ rather than tools for involvement in their care, and as such may not have used YCNY to its full potential. One ward substantially implemented flexible components and factors such as strong leadership on the part of the ward manager and a sense of distributed ownership across the ward may have facilitated this.

There is little evidence available on the benefits and challenges of a hybrid intervention approach. This study suggests there may be benefits of allowing intervention components to be flexibly tailored to the local context. Birch 2 successfully implemented flexible components, and it may be that the opportunity to devise components facilitated a sense of autonomy among staff and allowed for a deeper understanding of the intervention. Alternatively, Birch 2 may have possessed a pre-existing appetite for culture change and multidisciplinary team working that facilitated the implementation of flexible components. Future hybrid interventions may benefit from one or two highly motivated facilitators, together with widespread training of other relevant staff, in order to allow for strong leadership as well as a sense of distributed ownership. Further, YCNY appeared to resonate with wards with an interest in mobility/rehabilitation (such as Birch 2) and flexible components may best thrive in environments where the ethos of the intervention fits that of the setting and allows staff to achieve pre-existing goals. It may be that interventions focused on involvement are more likely to be successful in such settings due to a pre-existing culture of involvement. Similarly, the acuity level of the patients impacted patients’ engagement with YCNY and the extent to which staff believed involvement was a desirable goal. As such, how contextual factors such as ward setting influence attitudes around involvement is likely to impact on the success of hybrid involvement interventions.

The majority of wards, however, did not substantially implement flexible components. Flexible components seemed to be in conflict with the taskified nature of staff’s roles.^5 7^ The repetitive, task-based nature of some clinical roles may leave little space for the kind of reflective thinking needed for devising flexible components. Further, the fixed components were often delivered in a taskified manner, with staff viewing giving out the booklet as another task to hurry through, rather than a prompt for enhancing communication. While the booklet was designed as a tool to enact behaviour change, it was often merged into other ‘information’ and rarely used as such a tool. It is unclear how, and to what extent, the booklet facilitated the four key functions. The booklet prompts patients to consider ways to be involved in the four functions (eg, moving around the ward, asking staff about any changes to medications) and to use the examples as prompts to consider their own needs. However, due to a lack of time, staff often did not frame the booklet as a relational involvement tool to patients and this may have meant that the booklet was used by patients in a more instrumental manner.

As such, the provision of fixed components may have contributed to the taskification of the intervention. Previous research into the delivery of a complex patient experience intervention found that, while having a participatory role in the creation of the intervention sometimes enhanced staff engagement, facilitation of the intervention largely fell to action researchers rather than healthcare staff due to service pressures.^19^ Together with our findings, this suggests that expecting busy staff to engage creatively in complex interventions may be unrealistic without significant external support. YCNY requires staff to facilitate intervention delivery following an initial training session; however, flexible component implementation may work best with greater mentorship and support. While YCNY was designed with the intention that staff would have support from volunteers, this did not occur largely due to COVID-19 restrictions. The trial was not able to provide funding to support NHS staff to develop and deliver YCNY components, and this may have been a barrier to staff finding the time to fully engage with YCNY.

Taskification of an intervention may be especially problematic for interventions targeting involvement. Patient involvement requires communication between patients and staff, and interventions which become task based may not allow for the level of staff and patient engagement required to enact involvement. Patient involvement is not always an intuitive idea for patients nor staff. There is much between-patient and within-patient variability in the extent to which patients desire involvement.^20^ Furthermore, there are numerous barriers to enacting involvement, including staff and patients’ expectations of their roles and entrenched hospital practices.^21^ As such, enabling involvement requires encouraging staff and patients to think differently about patients’ roles, and delivering fixed intervention components in a task-based manner may be insufficient to encourage such a mindset shift. In this manner, the design of YCNY’s fixed components may have been insufficient to encourage involvement in its diverse forms.

One further complication of delivering YCNY was the randomised controlled trial context. Staff could become confused between the processes of YCNY and the trial. For example, the roles of the research nurses and ward staff sometimes became confused, with research nurses delivering aspects of the intervention (eg, giving out booklets) and ward staff identifying patients for the trial. This confusion around staff roles may have diminished staff engagement in YCNY, making it less likely that staff would take ownership and devise flexible components. As such, the implementation of hybrid interventions within the context of a trial may be especially challenging. This issue may be especially apparent in cluster randomised controlled trials and may indicate that research nurses could benefit from training in cluster randomised controlled trial methodology, given that they will be most familiar with randomisation at an individual level.

### Limitations

Limitations include the lack of a diverse and representative sample of patients. While patient ethnicity for the process evaluation was similar to that of the PACT trial (for the eight wards participating in the process evaluation, 97% of participants recruited into the PACT trial nested study were of white ethnicity), the lack of ethnic diversity is a limitation. Patients tended to consider themselves mobile and independent and as such may not have been those most appropriate for the intervention. Recruitment challenges impeded data collection including ward closures and high staff leave due to the COVID-19 pandemic. YCNY was designed to include family members/carers, and family members/carers of those with dementia may have particularly valuable perspectives on their role in engaging with YCNY; however, the perspectives of family members/carers were not gained due to recruitment difficulties. It is worth noting that the observations were relatively light touch and due to practical constraints, the researcher could not become immersed within the ward setting. The observations provided valuable data on the visibility of YCNY materials and interactions between patients and staff. However, the researcher may have lacked access to day-to-day interactions that may have provided more nuance around care provision and this may have inflated their sense of the extent to which YCNY had become taskified. Finally, the process evaluation only reports on YCNY implementation on eight wards and may not be representative of implementation across all wards taking part in the PACT trial.

## Conclusions

Hybrid interventions consisting of both fixed and flexible components are thought to allow for adaptation to the local context while maintaining fidelity. However, within a stretched healthcare system, staff may lack the time to engage in the creative thinking required to develop and implement flexible components. Furthermore, the provision of fixed components may risk that staff deliver these components in a routine, taskified manner, and interventions targeting culture change around patient involvement may be especially at odds with this taskified approach. Findings from the present study suggest that flexible components are challenging to implement and may require strong leadership, a team-wide approach and a culture of openness to change in order to be successful. Complex intervention delivery within pressurised healthcare settings may benefit from additional support to bolster the role of healthcare staff. All in all, research involving hybrid interventions appears to be at a crossroads, and how best to approach the implementation of fixed and flexible intervention components within a stretched system is an issue that warrants further attention.

## supplementary material

10.1136/bmjqs-2024-017268 online supplemental file 1

## Data Availability

Data are available upon reasonable request.
