# Supplementary material for: Understanding the challenges and successes of implementing ‘hybrid’ interventions in healthcare settings: findings from a process evaluation of a patient involvement trial
Source: BMJ Qual Saf. 2024 Aug 6;34(2):e017268. doi: 10.1136/bmjqs-2024-017268 (PMC11874326; doi:10.1136/bmjqs-2024-017268)
Supplement: online supplemental file 1 [file bmjqs-34-2-s001.pdf]

Baseline Interview Guide: Patient

Section 1: Getting to know people

- Find out about their life e.g. where they live, who they live with, if they have any family etc.

When they're not in hospital:

- How is their health generally?
- How are they managing their medication (getting them, taking them, understanding them)?
- How are they managing with normal daily activities (e.g. washing, dressing, going to the toilet, getting around, shopping, seeing friends and family)?
- Any problems with mobility or falls?
- Can they get help and support when they need it? If so, who provides it?

Section 2: Being admitted to hospital

- Why have they been admitted to hospital? How did they come to be in hospital?
- How do they feel about being in hospital?
- What do they think will happen next?
- How do they feel about going home? What issues do they think they could face when they leave hospital to go home?

Section 3: Questions about their health and care

- Do they feel they have a good understanding of their health and the treatment they are receiving?
- How involved have they been so far in discussions about them and their treatment and care? (How do they feel about this? Probe for: *choice*, decision-making, *information (given and received)*, consultation about discharge process?)
- How involved have they been able to be in their self-care whilst in hospital (e.g. normal daily activities, taking medication)? (How do they feel about this?)

Post-Discharge Interview Guide: Patient

1. Managing everyday life post-discharge

- How do you feel about being back at home?
- How is your health at the moment?
- To what extent is life 'back to normal'?
- How are you managing your medication (getting them, taking them, understanding them)?
- How are you managing with normal daily activities (e.g. washing, dressing, going to the toilet, getting around, shopping, seeing friends and family)?
- Any problems with walking around or falls?
- Can/could you get help and support if/when you need it? If so, who provides it?

2. Usage of the YCNY intervention

- Which components of the YCNY intervention were received in hospital and how were they delivered? (Booklet, Video, Posters, Advice sheet)
- How did you use the YCNY intervention in hospital and at home? (Booklet, Video, Posters, Advice sheet)
- What do you think about the YCNY intervention? Was it helpful?
- How did the YCNY intervention support:
    - preparation for discharge
    - decision-making, communication with staff
    - know more about health/medications/how to get up and moving
    - family/carers to be involved
- The role of hospital staff and volunteers in supporting use of the YCNY intervention
- The role of family/carers in supporting use of the YCNY intervention
- Was there anything that helped or hindered using the YCNY intervention

Topic guide for staff interviews

- What is your staff role? (How long since you qualified? How long have you been working on this ward?)
- How have you supported the YCNY intervention?
- What is your understanding of the YCNY intervention (process and ultimate aims)?
- What was delivered on your ward and who was involved in its delivery?
- Have you been able to implement any of the changes that you planned?
- What are your views on the training provided in support of the YCNY intervention?
- How successful do you think the YCNY intervention has been on your ward?
- How might the YCNY intervention have
    i. changed the way staff think about discharge and helped them involve patients/carers in their care; altered discharge planning
    ii. empowered patients and helped them prepare for discharge
- Any facilitators or barriers that may have helped or hindered staff delivering the YCNY intervention and/or patients using it?
- How does YCNY align with organisational priorities?
